# Impact of Prolonged Mechanical Ventilation on Ferroptosis in Renal Ischemia/Reperfusion Injury in Rats

**DOI:** 10.1155/2020/6097516

**Published:** 2020-02-22

**Authors:** Fangfang Zhou, Yi Yang, Lianxin Luo, Yixin Chen, Qun Luo, Jianghua Chen

**Affiliations:** ^1^Kidney Disease Center, The First Affiliated Hospital, College of Medicine, Zhejiang University, Hangzhou, Zhejiang, China; ^2^Department of Nephrology, HwaMei Hospital, University of Chinese Academy of Sciences, Ningbo, Zhejiang, China

## Abstract

We here investigated the impact of mechanical ventilation (MV) time on ferroptosis in a rat renal ischemia/reperfusion injury (IRI) model. Thirty-two male adult Sprague Dawley rats were divided into four groups (*n* = 8/group): the sham group, IRI group, IRI+MV-4 h group, and IRI+MV-12 h group. Rats in the IRI group were subjected to 45 min bilateral renal ischemia. Rats in the IRI+MV groups were additionally mechanically ventilated with tracheal intubation after 45 min bilateral renal ischemia. Morphological changes associated with kidney injury and ferroptosis were assessed by hematoxylin and eosin staining and electron microscopy. Levels of the central regulator of ferroptosis, glutathione peroxidase 4 (GPX4), and lipid peroxidation markers 4-hydroxynonenal (4HNE) and superoxide dismutase 2 (SOD2) were determined in the kidney tissue by western blotting. Glutathione (GSH) levels were assessed in the serum and kidney homogenate. Scr levels in the IRI+MV-12 h group were significantly higher than those in the sham, IRI, and IRI+MV-4 h groups (all *P* < 0.001). Electron microscopy revealed the most pronouncedly abnormal mitochondrial morphology, suggestive of ferroptosis, in the IRI+MV-12 h group. The GPX4 and SOD2 protein levels progressively decreased in the following order: sham group > IRI group > IRI+MV-4 h group > IRI+MV-12 h group (*P* < 0.05 for all comparisons). By contrast, the 4HNE levels progressively increased in the kidney, with the highest values in the IRI+MV-12 h group (*P* < 0.05, vs. the IRI group and vs. the IRI+MV-4 h group). Further, the GSH levels in the serum and kidney homogenates were significantly reduced in the IRI+MV-12 h group (*P* < 0.01, vs. IRI group and vs. the IRI+MV-4 h group). A significant positive correlation was observed between the serum and kidney GSH levels (*r*^2^ = 0.542, *P* = 0.03). These observations suggested that prolonged MV may exacerbate renal function failure, already initiated by IRI, by ferroptosis. Depletion of GSH may contribute to this effect, which requires further investigation.

## 1. Introduction

Mechanical ventilation (MV) is associated with acute kidney injury (AKI) in the intensive care unit [1, 2]. Our previous perspective observational study and other studies revealed that extubation delay is one of the independent risk factors for AKI development in a cardiac surgical patient. Further, hemodynamic changes, neurohormonal modifications, and systemic inflammation induced by MV may contribute to the development of AKI [3, 4]. However, the understanding of the causal relationship between MV and subsequent AKI is incomplete.

Increased production of reactive oxygen species (ROS) in response to mechanical stress has been described in a variety of cell types [5, 6]. Of note, Chiang, et al. [6] demonstrated that MV with high tidal volume increases the generation of ROS, which acts as an initiating signal in the lung epithelium in response to increased cellular stretch. This suggests that ROS may play an important role in the pathogenesis of ventilator-induced lung injury.

Research into oxidative cell death has revealed new mechanisms of cell death, one of which is ferroptosis [7]. Ferroptosis was recently discovered as a nonapoptotic cell death, distinct from other cell death pathways, such as apoptosis, necrosis, and pyroptosis [8]. This form of regulated cell death is involved in various conditions that lead to AKI, e.g., folic acid-induced injury and ischemia/reperfusion injury (IRI) [9, 10]. Unlike apoptosis and necroptosis, there are no known receptors that trigger ferroptosis; rather, it is triggered by glutathione (GSH) depletion or inactivity of glutathione peroxidase 4 (GPX4), which results in an accumulation of lethal levels of lipid ROS [11, 12]. The GSH-GPX4 axis is the sole known cellular system responsible for the efficient repair of oxidized phospholipids. According to *in vivo* studies, regulation of ferroptosis is a key function of GPX4 [7].

For a pathological perspective, mediators of inflammation or immunomediated factors related to primary lung pathology can also damage the kidney. For example, Hegeman et al. [13] demonstrated that MV induces endothelial activation and inflammation in a time-dependent manner, not only in the lung but also in distal organs, such as the kidney. Hence, we here did the study to see if prolonged MV enhanced ferroptosis in the kidney following IRI, resulting worsen renal function.

## 2. Materials and Methods

### 2.1. Animals

Male Sprague Dawley rats (SPF, *n* = 32; weighing 240–320 g) were obtained from Shanghai SLAC Laboratory Animal Co., Ltd. (Licence No. SCXK(HU)2017-0005). The animals were maintained in a humidity and temperature-controlled environment, with a 12 h light-dark cycle, with unlimited access to food and water. The experiments were performed in accordance with Chinese legislation regarding the use and care of laboratory animals and were approved by the Animal Care and Use Committee of Ningbo University (China).

### 2.2. Rat Model of Renal IRI

Rats were randomly assigned to one of four experimental groups (*n* = 8/group): sham group, subjected to sham operation; IRI group, subjected to 45 min bilateral renal ischemia; IRI+MV-4 h group, subjected to 45 min bilateral renal ischemia and 4 h MV; and IRI+MV-12 h group, subjected to 45 min bilateral renal ischemia and prolonged 12 h MV.

The rats were anesthetized with 10% chloralic hydras (3 mL/kg weight), administered intraperitoneally. Additional anesthesia was used when necessary. A midline incision was made using a sterile scalpel, and the skin and peritoneum were separated layer by layer to reach the abdominal cavity. The renal pedicles were exposed and clamped bilaterally for 45 min with microvascular clamps. Subsequently, the clamps were removed to allow reperfusion of the kidneys. Renal color change was monitored to confirm blood reflow before suturing the incision. During the operation, saline was provided to keep the rats fully hydrated. After the operation, the abdominal cavity was closed by layered suture. For the sham-operated group, the animals were treated as above except for the renal pedicle clamping. After 12 h reperfusion, all rats were euthanized, and the blood (0.5 mL) and kidneys were collected.

Rats from the IRI+MV-4 h and IRI+MV-12 h groups were additionally mechanically ventilated with tracheal intubation after 45 min bilateral renal ischemia. The animals were ventilated using a volume-driven small animal ventilator (HX-101E, Techman Software, Chengdu, China) for 4 h or 12 h, as appropriate. The tidal volume was set at 7 mL/kg body weight. The respiratory rate was set at 40 breaths/min. The inspiration to expiration ratio was set at 1 : 2. Breathing air was humidified and enriched with oxygen. The tail vein was cannulated for continuous infusion of isotonic saline at a rate of 10 mg/kg/h. Body temperature was maintained at 37°C by using a heating blanket. The sham and IRI groups did not undergo MV.

### 2.3. Histopathological Evaluation of the Kidney

The kidneys were fixed in 4% paraformaldehyde solution, embedded in paraffin, sectioned (4 *μ*m thickness), and stained with hematoxylin and eosin (HE). Renal tubular injury was evaluated using an Olympus BX-43 light microscope. Tubular injury was defined as the presence of tubular casts, brush border loss, flattened epithelium, and/or sloughing of tubular epithelial cells, which were graded according to the semiquantitative scores developed by Paller and Neumann [14]as follows: (1) tubular epithelial smoothness or tubular expansion—score 1, (2) loss of brush-like edge—score 1 or 2, (3) obstruction of tubular lumen—score 1 or 2, (4) cytoplasmic vacuolization—score 1, and (5) cell necrosis—score 1 (the higher the score, the more severe the damage). The highest achievable score was seven.

### 2.4. Transmission Electron Microscopy

A tissue block of approximately 1 mm^3^ was collected from each kidney. The tissue was fixed in 4% paraformaldehyde and postfixed in 1% osmium tetroxide. The specimens were then washed in 0.1 M phosphate-buffered saline, dehydrated in graded alcohols and acetone, and embedded in araldite resin. Ultrathin sections (120 nm thickness) were cut and placed on copper grids. The sections were stained with 4% uranyl acetate and lead citrate. Transmission electron microscopic examination was performed using a Philips TECNA-10 electron microscope (Philips, Eindhoven, The Netherlands). The mitochondrial area was determined by using ImageJ software.

### 2.5. Biochemical Analysis

The blood was centrifuged (3000 × *g* for 10 min). The serum was collected and stored at 4°C, and the kidney tissue (weighed 0.05 g) was homogenized by a homogenizer (SCIENTZ-48, Ningbo, China) with 9 volumes (1 : 9, *w*/*v*) of ice-cold normal saline. The kidney homogenate was centrifuged at 3000 × *g* for 10 min at 4°C, and the resultant supernatant was used for biochemical analysis. The levels of serum creatinine (Scr) and GSH were determined by means of chemical chromatometry using relevant assay kits according to the manufacturers' instructions as follows.

Serum GSH levels were determined with a GSH + GSSG assay kit (S0053, Beyotime Biotechnology, China). According to the instruction of the manufacturer, total glutathione (GSH + GSSG) was assayed using the 5,5-dithio-bis (2-nitrobenzoic) acid- (DTNB-) GSSG reductase recycling. GSSG was measured by measuring 5-thio-2-nitrobenzoic acid (TNB) which was produced from the reaction of reduced GSH with DTNB. The rate of TNB formation was measured at 412 nm on a UV spectrometer (SpectraMax Plus 384 MD, USA). Levels of T-GSH and GSSG were obtained from linear standard curves. The GSH level was calculated according to the following equation: GSH = T‐GSH − 2 × GSSG.

Scr levels were determined using a creatinine kit based on sarcosine oxidase (C011-2, Jiancheng Biotechnology, Nanjing, China). The procedural details are as follows: (i) Three sterile Eppendorf tubes were designated as a blank tube, which was supplemented with 6 *μ*L of ddH_2_O; a standard tube, which was supplemented with 6 *μ*L of a standard specimen; and a sample tube, which was supplemented with 6 *μ*L of a serum sample. (ii) A volume of 180 *μ*L of enzyme solution A was added to each tube. (iii) The samples were fully mixed and incubated at 37°C for 5 minutes. (iv) Absorbance at 546 nm was measured for all of the tubes using the UV spectrometer (SpectraMax Plus 384 MD, USA), and the values were defined as A1. (v) Following the method of absorbance measurement, a blank tube, which was supplemented with 6 *μ*L of ddH_2_O, and a standard tube were weighed and incubated at 37°C for 5 minutes. (vi) Absorbance at 546 nm was again measured for all of the tubes and defined as A2. (vii) Scr concentration was calculated using the following equation: concentration = [(detected A2‐*K* × detected A1) − (blank A2‐*K* × blank A1)]/[(standard A2‐*K* × standard A1) − (blank A2‐*K* × blank A1)] × standard substance concentration, where standard substance concentration is 50 *μ*mol/L and dilution factor *K* is 0.76.

### 2.6. Western Blotting

The kidney tissue (weighed 0.1 mg) was homogenized in 500 *μ*L RIPA lysis buffer (P0013B, Beyotime Biotechnology, China). Total protein was extracted from the supernatants after centrifugation at 11,000 × *g* for 20 min at 4°C. The protein content was determined using Bradford protein assay. Proteins were separated by polyacrylamide gel electrophoresis (SDS-PAGE) and transferred to polyvinylidene difluoride membrane (IPVH00010, EMD Millipore, Billerica, MA, USA). The membrane was blocked in 5% bovine serum albumin at room temperature for 1 hour and probed overnight at 4°C with primary antibodies specific for GPX4 (ab125066, Abcam, Cambridge, UK, 1 : 1000), superoxide dismutase 2 (SOD2) (13141S,CST, Boston, MA, 1 : 1000), and 4-hydroxynonenal (4HNE) (ab46545, Abcam, Cambridge, UK, 1 : 2000). Primary antibodies were diluted by QuickBlock™ Primary Antibody Dilution Buffer for Western Blot (P0256, Beyotime Biotechnology, China). The blots were then incubated at room temperature for 2 hours with goat anti-rabbit or anti-mouse secondary antibodies (both diluted at 1 : 5000), as appropriate. Protein bands were visualized and scanned using a ChemiDoc XRS+ Imaging System (Bio-Rad, Inc., USA). Blot images were acquired for quantification using a digital imager and analyzed with imaging software.

### 2.7. Statistical Analysis

Data are presented as the mean ± SEM. Comparisons of dependent variables between the groups were made by a one-way analysis of variance (ANOVA), followed by Tukey's multiple comparison test as a post hoc test to compare the group means. The relationships between serum GSH levels and kidney GSH levels were evaluated using Spearman's rank correlation test. The significance was established at *P* < 0.05. Statistical analysis was not performed for electron microscopy images because of the small sample size (*n* = 3/group). All statistical analyses were performed using IBM SPSS statistic 25 (IBM, Armonk, NY) and PRISM version 4.0 (GraphPad Software, Inc., La Jolla, CA).

## 3. Results

### 3.1. Changes in the Renal Function in Each Group

**Figure 1:**
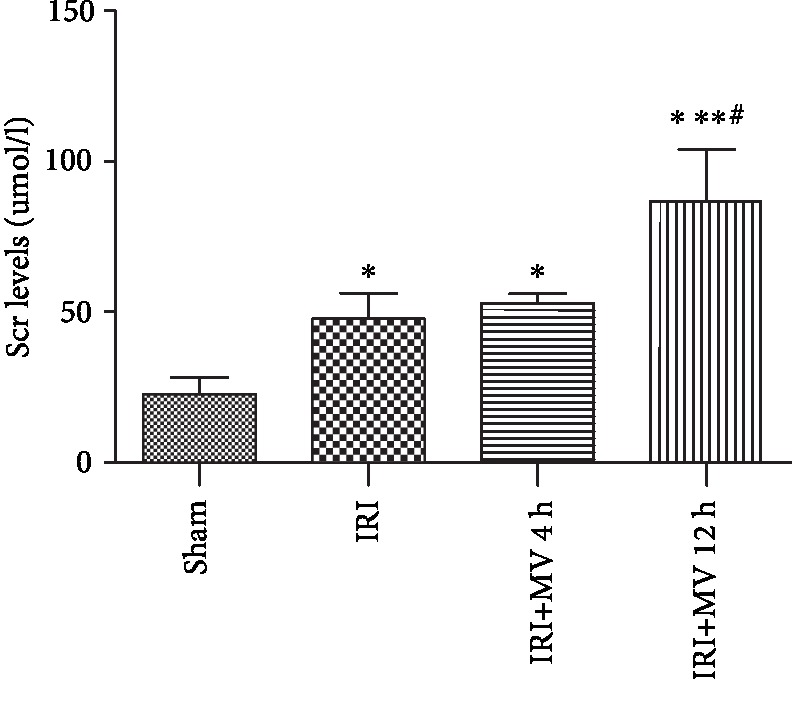
Evaluation of kidney function (serum creatinine (Scr)) from rats in the sham group, IRI group, IRI+MV-4 h group, and IRI+MV-12 h group. The results are presented as the mean ± S.D. Sham group: sham operation; IRI group: subjected to 45 min bilateral renal ischemia; IRI+MV-4 h group: subjected to 45 min bilateral renal ischemia and underwent 4-hour MV; IRI+MV-12 h group: subjected to 45 min bilateral renal ischemia and underwent 12-hour MV. GSH: glutathione; IRI: ischemia-reperfusion injury; MV: mechanical ventilation. ^∗^*P* < 0.001, vs. sham; ^∗∗^*P* < 0.001, vs. IRI; ^#^*P* < 0.001, vs. IRI+MV-4 h.

The Scr levels in the IRI+MV-12 h group (86.74 ± 17.18 *μ*mol/L) were significantly higher than those in the sham (22.65 ± 5.60 *μ*mol/L), IRI (47.67 ± 8.58 *μ*mol/L), and IRI+MV-4 h (52.80 ± 3.28 *μ*mol/L) groups (all *P* < 0.001). However, the Scr levels showed no significant improvement in the IRI and IRI+MV-4 h groups (*P* = 0.397) (Figure 1). These observations suggested that prolonged MV may exacerbate renal function failure, already initiated by IRI.

### 3.2. Renal Histopathological Examination

**Figure 2:**
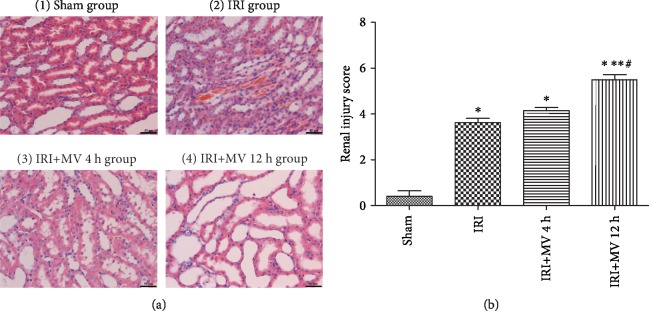
(a (1–4), b) Pathological morphology observation and scoring of renal injury from rats in the Sham group (1), IRI group (2), IRI+MV-4 h group (3), and IRI+MV-12 h group (4). The results are presented as the mean ± S.D. Sham group: sham operation; IRI group: subjected to 45 min bilateral renal ischemia; IRI+MV-4 h group: subjected to 45 min bilateral renal ischemia and underwent 4-hour MV; IRI+MV-12 h group: subjected to 45 min bilateral renal ischemia and underwent 12-hour MV. IRI: ischemia-reperfusion injury; MV: mechanical ventilation. ^∗^*P* < 0.05, vs. sham; ^∗∗^*P* < 0.05, vs. IRI; ^#^*P* < 0.06, vs. IRI+MV-4 h.

**Figure 3:**
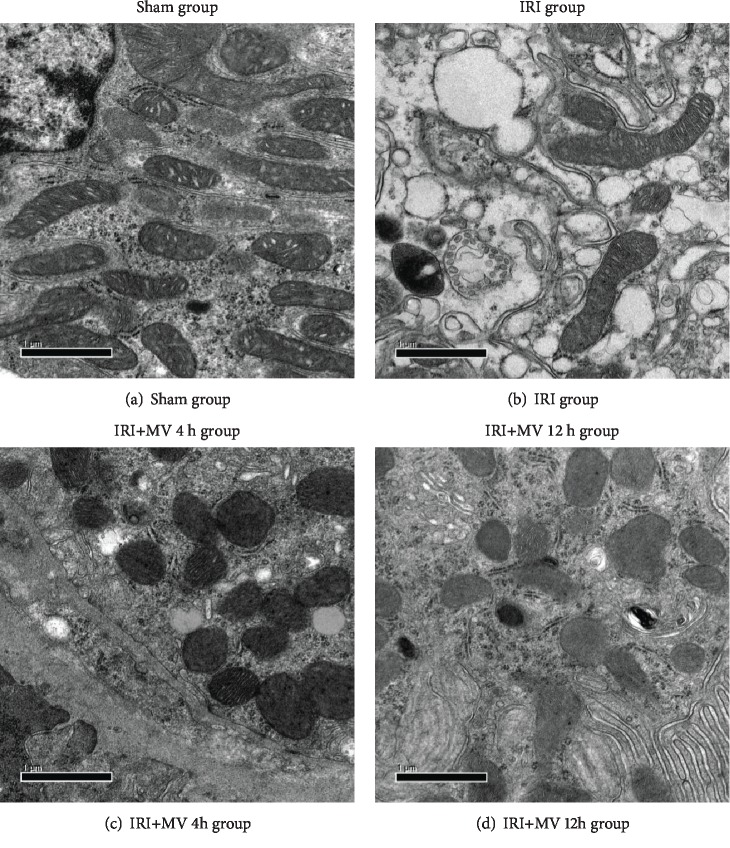
(a–d) Representative electron micrographs of rats in the sham group (a), IRI group (b), IRI+MV-4 h group (c), and IRI+MV-12 h group (d). Sham group: sham operation; IRI group: subjected to 45 min bilateral renal ischemia; IRI+MV-4 h group: subjected to 45 min bilateral renal ischemia and underwent 4-hour MV; IRI+MV-12 h group: subjected to 45 min bilateral renal ischemia and underwent 12-hour MV. IRI: ischemia-reperfusion injury; MV: mechanical ventilation.

In the sham group, the renal tissue sections exhibited normal morphology, as determined by HE staining [Figure 2(a) (1)]. Histological examination of the kidneys exposed to IRI [Figure 2(a) (2)], IRI with 4 h MV [Figure 2(a) (3)], and IRI with 12 h MV [Figure 2(a) (4)] revealed a distinctive pattern of ischemic renal injury, characterized by a widespread degeneration of the tubular architecture, tubular dilatation, swelling, congestion, increased interstitial edema, brush border loss, and thinning and/or flattening of the tubular epithelium. Furthermore, the pathological scores of renal tubule injury in the IRI+MV-12 h group were significantly higher than those in the IRI and IRI+MV-4 h groups (*P* < 0.05) (Figure 2(b)). However, there was no significant difference between the scores for the IRI and IRI+MV-4 h groups (*P* > 0.05). In addition, electron microscopy analysis revealed the most abnormal mitochondrial morphology indicative of ferroptosis in the IRI+MV-12 h group, i.e., the presence of relatively small mitochondria with reduced and/or absent cristae (Figure 3(d)).

### 3.3. Western Blotting Analysis of Specific Protein Levels in Each Group

**Figure 4:**
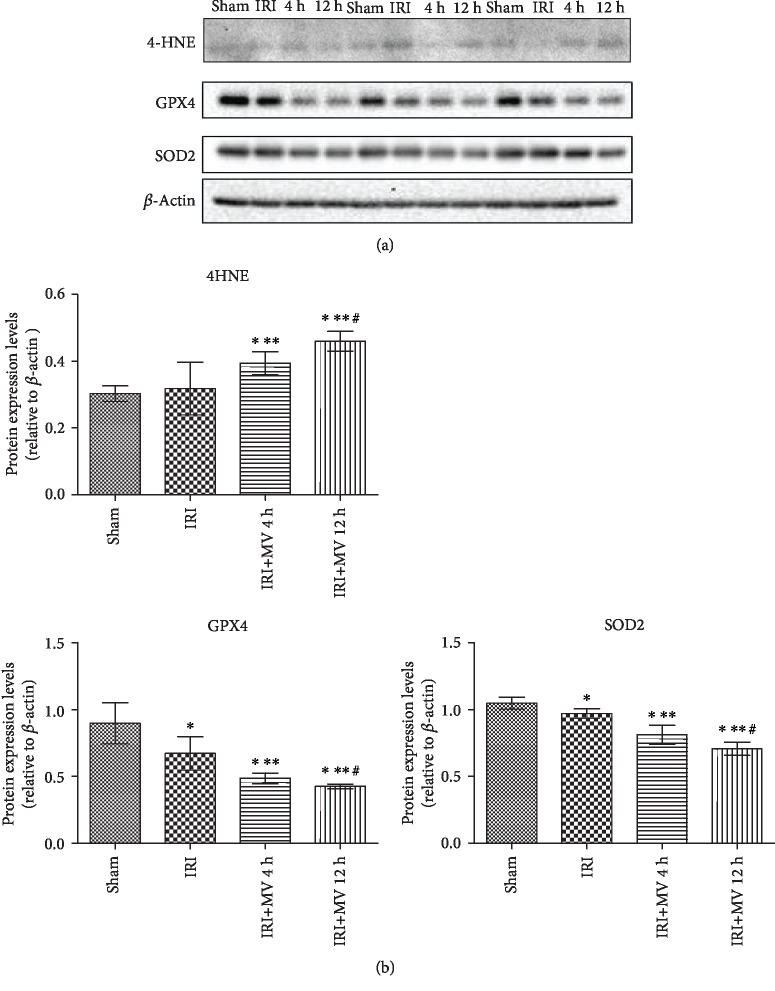
(a, b) Representative western blots and quantitative evaluation of 4HNE, GPX4, and SOD2 expression in kidney tissue from rats in the sham group, IRI group, IRI+MV-4 h group, and IRI+MV-12 h group. The results are presented as mean ± S.D. Sham group: sham operation; IRI group: subjected to 45 min bilateral renal ischemia; IRI+MV-4 h group: subjected to 45 min bilateral renal ischemia and underwent 4-hour MV; IRI+MV-12 h group: subjected to 45 min bilateral renal ischemia and underwent 12-hour MV. 4HNE: 4-Hydroxynonenal; GPX4: glutathione peroxidase 4; SOD2: superoxide dismutase 2; IRI: ischemia-reperfusion injury; MV: mechanical ventilation. ^∗^*P* < 0.05, vs. sham; ^∗∗^*P* < 0.05, vs. IRI; ^#^*P* < 0.05, vs. IRI+MV-4 h.

To verify the effect of prolonged MV on ferroptosis in the IRI kidney, we analyzed protein levels in the kidney by western blotting. The analysis revealed that the levels of GPX4, a ferroptosis marker, were progressively reduced in the order sham group > IRI group > IRI+MV-4 h group > IRI+MV-12 h group, with the lowest level in the latter, achieving statistical significance (*P* < 0.05 for all comparisons). Lipid peroxidation (determined based on 4HNE levels), which follows ferroptosis, progressively increased in the kidney from 0 h MV up to 12 h MV, with the highest levels in the IRI+MV-12 h group, achieving statistical significance in comparison with the IRI group and IRI+MV-4 h group (*P* < 0.05). By contrast, the levels of SOD2, an antioxidant enzyme, progressively declined from 0 h MV to 12 h MV, in a time-dependent manner (both *P* < 0.05, vs. the IRI group and vs. the IRI+MV-4 h group) (Figures 4(a) and 4(b) and Table 1).

### 3.4. Serum and Kidney GSH Levels in Each Group

**Figure 5:**
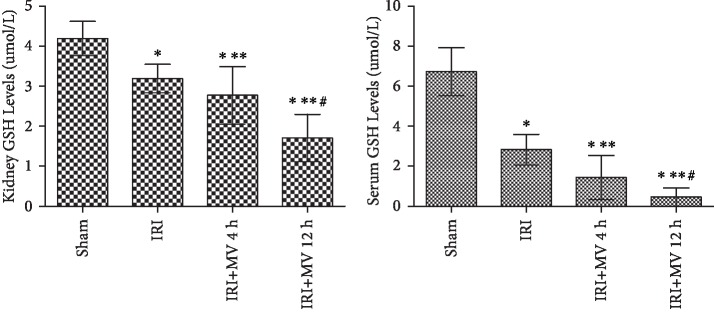
GSH levels in serum and kidney homogenate from rats in the sham group, IRI group, IRI+MV-4 h group, and IRI+MV-12 h group. The results are presented as the mean ± S.D. Sham group: sham operation; IRI group: subjected to 45 min bilateral renal ischemia; IRI+MV-4 h group: subjected to 45 min bilateral renal ischemia and underwent 4-hour MV; IRI+MV-12 h group: subjected to 45 min bilateral renal ischemia and underwent 12-hour MV. GSH: glutathione; IRI: ischemia-reperfusion injury; MV: mechanical ventilation. ^∗^*P* < 0.01, vs. sham; ^∗∗^*P* < 0.01, vs. IRI; ^#^*P* < 0.01, vs. IRI+MV-4 h.

**Figure 6:**
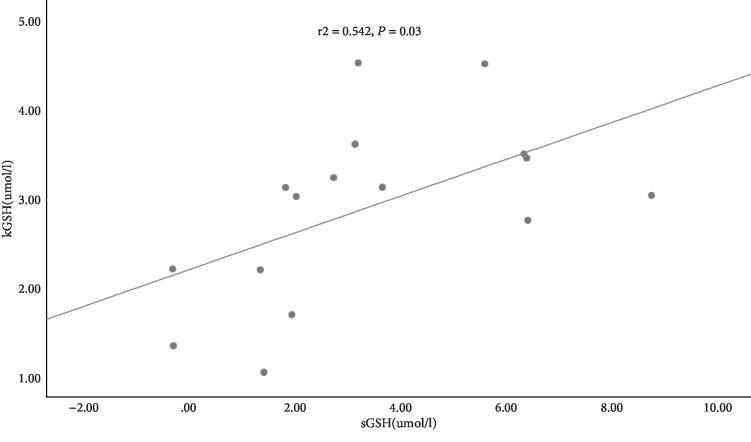
The correlation between serum and kidney GSH levels (*r*^2^ = 0.542, *P* = 0.03) from rats. kGSH: kidney glutathione; sGSH: serum glutathione.

GSH depletion triggers ferroptosis. The GSH levels in both serum and kidney homogenates were significantly reduced in the IRI+MV-12 h group and achieved statistical significance in comparison with other groups (both *P* < 0.01, vs. the IRI group and vs. the IRI+MV-4 h group) (Figure 5 and Table 2). A significant positive correlation was noted between the serum and kidney GSH levels (*r*^2^ = 0.542, *P* = 0.03) (Figure 6).

Collectively, the results of our study indicated that a prolonged MV treatment might play a role in promoting ferroptosis in the IRI rat model. Depletion of GSH after prolonged MV might contribute to this effect, which requires further investigation.

## 4. Discussion

The most important finding of the current study was that prolonged MV may exacerbate renal function failure, already initiated by IRI, by ferroptosis. As demonstrated by elevated Scr levels and an increased pathological score of the renal tubule injury following 12 h MV, prolonged MV exacerbated kidney injury in the *in vitro* model of IRI. As opposed to 4 h MV or IRI injury alone, GPX4 levels decreased in a time-dependent manner, which was accompanied by elevated 4HNE levels and reduced SOD2 levels during 12 h MV. This suggested that prolonged MV might increase the sensitivity of the kidney exposed to IRI to ferroptosis. Furthermore, we observed a significant positive correlation between the serum and kidney GSH levels. This requires further study into the causal connection between the systemic GSH depletion and renal ferroptosis.

A meta-analysis revealed that invasive MV is associated with a threefold increase in the odds of developing AKI [1]. Strong evidence exists in support of the various potential mechanisms underlying ventilator-induced kidney injury (VIKI), e.g., involving hemodynamic changes, neurohormonal modifications, and systemic inflammatory mediators generated by a ventilator-induced lung injury. Nevertheless, the knowledge regarding the mechanisms of VIKI remains limited.

Further, convincing evidence exists linking the redox imbalance and MV [15]. GSH is often used as an indicator of cellular redox status. GSH is a tripeptide cellular antioxidant that protects the lipids, DNA, and proteins from oxidative damage [16]. Cellular GSH is typically depleted upon redox imbalance [17, 18]. Pires et al. [19] reported a significant reduction of GSH levels in the lung homogenates of MV group animals in comparison with the levels in a spontaneous respiration group. Further, according to recent studies, MV-induced oxidative stress can affect other targets besides the lung. It has been demonstrated that prolonged MV leads to a significant depletion of diaphragmatic GSH, therefore promoting oxidative stress in the diaphragm [20, 21]. Similarly, in the present study, we showed that the GSH levels in the serum and kidney homogenates were significantly reduced in the IRI+MV-12 h group. In this regard, it is possible that prolonged MV induces mechanical stress, leading to systemic oxidative stress, which may be one of the crucial factors in the pathogenesis of VIKI.

Further, cellular GSH plays a key protective role in the suppression of ferroptosis [22, 23]. Ferroptosis is an oxidative form of regulated cell death, associated with the accumulation of lipid ROS because of enhanced lipid peroxidation. It is most likely triggered by the failure of GSH-dependent antioxidant defenses, not receptors, which differs from what is observed in other forms of cell death [24, 25]. Therefore, we speculate that ferroptosis occurs in the kidney once prolonged MV depletes GSH levels and contributes to a subsequent kidney injury.

GPX4 is the only enzyme capable of converting toxic lipid hydroperoxides to nontoxic lipid alcohols and uses GSH as the substrate during ferroptosis. The protein is the central regulator of ferroptosis. Genetic inactivation or pharmacological inhibition of GPX4 promotes ferroptotic cell death *in vitro* [26–28]. In addition, genetic ablation of GPX4 in mouse leads to increased accumulation of oxidized phospholipid products in the kidney, resulting in AKI and early mortality [29]. In the current study, we observed that the levels of key regulators of ferroptosis, such as GPX4 and GSH, were progressively reduced (in the order sham group > IRI group > IRI+MV-4 h group > IRI+MV-12 h group), followed by increased lipid peroxidation (increased 4HNE levels and reduced SOD2 levels) in the kidney. This indicated that ferroptosis may play a role in the mechanism of how prolonged MV exacerbates renal function failure, already initiated by IRI.

The current study had several limitations. First, a direct cause and effect relationship between prolonged MV and ferroptosis in the kidney with IRI had not been unambiguously demonstrated. While GSH depletion and lipid peroxidation are observed in the lung tissue when the duration of MV is prolonged [19, 20], it is unclear whether and how the GSH depletion connects the lung and the kidney and induces subsequent ferroptosis in the kidney. And whether other factors of MV, like tidal volume or positive end-expiratory pressure ventilation (PEEP), will alter the extent of IRI ferroptosis, helping linking the stretch of MV per se to this downstream effect should be investigated in future studies. Second, there are two major signaling pathways in ferroptosis [30, 31]. One is the GSH-GPX4 axis studied herein, and the other is iron overload. Whether iron overload is involved in the pathogenesis of ferroptosis associated with prolonged MV should be explored further. Third, the current study was performed using a rat model, and species differences between rat and human cannot be excluded. Further, the duration of prolonged MV in our study was limited to 12 h. Twelve-hour MV in rat is quite different from 7 d MV in a patient. In this respect, animal models are far from reflecting the high complexity of critical illness in human, which involves multiple risk factors. Human studies are also needed to evaluate the relevance of these results in MV patients. Nonetheless, from the current study, we suggest prolonged controlled MV be avoided once the patient's condition is stable, as currently recommended in the Guidelines for Perioperative Care in Cardiac Surgery Enhanced Recovery After Surgery Society Recommendations (section of Extubation Strategies, class IIa, level B-NR) [32].

## 5. Conclusions

In summary, we here showed for the first time that prolonged MV may exacerbate renal function failure, already initiated by IRI, by enhanced ferroptosis. Depletion of GSH may contribute to this effect. This requires further exploration into a possible crosstalk between the lung and the kidney.

## Figures and Tables

**Table 1:** Protein expression levels of 4HNE, GPX4, and SOD2 in each group (relative to *β*-actin).

Protein expression levels (relative to *β*-actin)	Sham	IRI	IRI+MV-4 h	IRI+MV-12 h
4-HNE	0.30 ± 0.03	0.32 ± 0.02	0.39±0.03^∗,∗∗^	0.43±0.04^∗,∗∗,#^
GPX4	0.89 ± 0.15	0.67 ± 0.13^∗^	0.49±0.04^∗,∗∗^	0.44±0.04^∗,∗∗,#^
SOD2	1.05 ± 0.05	0.97 ± 0.04^∗^	0.77±0.01^∗,∗∗^	0.71±0.05^∗,∗∗,#^

Representative western blots and quantitative evaluation of 4HNE, GPX4, and SOD2 expression in kidney tissue from rats in the sham group, IRI group, IRI+MV-4 h group, and IRI+MV-12 h group. The results are presented as the mean ± S.D. Sham group: sham operation; IRI group: subjected to 45 min bilateral renal ischemia; IRI+MV-4 h group: subjected to 45 min bilateral renal ischemia and underwent 4-hour MV; IRI+MV-12 h group: subjected to 45 min bilateral renal ischemia and underwent 12-hour MV. 4HNE: 4-Hydroxynonenal; GPX4: glutathione peroxidase 4; SOD2: superoxide dismutase 2; IRI: ischemia-reperfusion injury; MV: mechanical ventilation. ^∗^*P* < 0.05, vs. sham; ^∗∗^*P* < 0.05, vs. IRI; ^#^*P* < 0.05, vs. IRI+MV-4 h.

**Table 2:** GSH levels in serum and kidney homogenate from rats in each group.

	Sham	IRI	IRI+MV-4 h	IRI+MV-12 h
Serum GSH (*μ*mol/L)	6.73 ± 1.20	2.83 ± 0.77^∗^	1.44±1.10^∗,∗∗^	0.46±0.46^∗,∗∗,#^
Kidney GSH (*μ*mol/L)	4.19 ± 0.43	3.19 ± 0.36^∗^	2.77±0.72^∗,∗∗^	1.71±0.59^∗,∗∗,#^

GSH levels in serum and kidney homogenate from rats in the sham group, IRI group, IRI+MV-4 h group, and IRI+MV-12 h group. The results are presented as the mean ± S.D. Sham group: sham operation; IRI group: subjected to 45 min bilateral renal ischemia; IRI+MV-4 h group: subjected to 45 min bilateral renal ischemia and underwent 4-hour MV; IRI+MV-12 h group: subjected to 45 min bilateral renal ischemia and underwent 12-hour MV. GSH: glutathione; IRI: ischemia-reperfusion injury; MV: mechanical ventilation. ^∗^*P* < 0.01, vs. sham; ^∗∗^*P* < 0.01, vs. IRI; ^#^*P* < 0.01, vs. IRI+MV-4 h.

## Data Availability

The data used to support the findings of this study are included within the article.
